# Relationship between oxysterols and mild cognitive impairment in the elderly: a case–control study

**DOI:** 10.1186/s12944-016-0344-y

**Published:** 2016-10-10

**Authors:** Quanri Liu, Yu An, Huanling Yu, Yanhui Lu, Lingli Feng, Chao Wang, Rong Xiao

**Affiliations:** School of Public Health, Beijing Key Laboratory of Environmental Toxicology, Capital Medical University, No.10 Xitoutiao, You An Men Wai, Beijing, 100069 Fengtai District China

**Keywords:** Oxysterols, 27–hydroxycholesterol, Alzheimer’s disease, Mild cognitive impairment, MoCA, Aβ1-40, Aβ1-42

## Abstract

**Background:**

To investigate the relationship between oxysterols and mild cognitive impairment (MCI) in a matched case–control study.

**Methods:**

The plasma levels of four oxysterols, 27–hydroxycholesterol (27–OHC), 24S–hydroxycholesterol (24S–OHC), 7α–hydroxycholesterol (7α–OHC) and 7β–hydroxycholesterol (7β–OHC), were analyzed by High Performance Liquid Chromatography–Mass Spectrometry (HPLC–MS) and compared between 70 MCI patients and 140 matched controls with normal cognition. The odds ratio (OR) was calculated using logistic analyses to assess the association between oxysterols and MCI.

**Results:**

Compared with controls with normal cognition, plasma level of 27–OHC was significantly higher in MCI patients. Logistic analyses suggested high plasma level of 27–OHC was significantly associated with MCI even after multivariate adjustment (OR = 2.86, 95 % CI: 1.52 ~ 5.37).

**Conclusions:**

Our findings suggested that the increased plasma level of 27-OHC was associated with MCI, suggesting high plasma levels of 27-OHC may pay an important role in the development of MCI.

## Background

Alzheimer’s disease (AD) is the most commonly recognized cause of dementia by memory loss and other intellectual symptoms serious enough to affect daily life in the elderly [1]. It contributes to premature death of elders after being diagnosed for 3 to 9 years [2]. MCI is the pre–clinical stage of AD with gradual cognitive decline but no influence on daily life activities. It is accepted that early intervention in MCI including decreasing the risk factors is useful and therefore many studies have focused on this stage [3].

Substantial epidemiological and molecular evidence has indicated that hypercholesterolemia is an important risk factor for neurodegenerative diseases [4]. However, clinical studies by using statins to lower the cholesterol for preventive and therapeutic management of neurodegeneration did not show the effects [5]. In addition, cholesterol in the blood cannot pass the blood brain barrier into central nervous system (CNS) [6]. The above facts cannot support the role of high plasma level of cholesterol in AD or MCI.

Oxysterols including 27–OHC, 24S–OHC, 7α–OHC and 7β–OHC are the oxidized derivatives of cholesterol, which can not only pass the blood brain barrier [7] but also have cytotoxic and pro-apoptotic properties [8, 9]. 27–OHC is the most abundant circulating oxysterol mainly produced in the liver [10]. Previous studies have also demonstrated an influx of the 27-OHC from the circulation into the brain [11]. Despite the fact that cholesterol cannot pass the blood–brain barrier, hypercholesterolemia is linked to an increased risk of neurodegenerative conditions including AD [12, 13], in particular in midlife [14]. Since there is a close correlation between circulating cholesterol and 27-OHC [15], hypercholesterolemia seems to result in an increased uptake of 27-OHC. Meanwhile, Heverin et al. [16] has demonstrated that treatment of mice with dietary cholesterol causes significant memory impairment and 27-OHC mediates the negative effects of dietary cholesterol on cognition. Therefore, there is possibility that 27-OHC is linking the excessive diet cholesterol or hypercholesterolemia and neurodegenerative conditions. A recent study showed a significant accumulation of 27–OHC in the brain of patients with AD [17]. In addition, one animal study showed the level of 27–OHC in 12–month–old rats is higher than that in 8–month–old rats, suggesting the accumulation of 27–OHC in the brain with age [18].

Despite the accumulated evidence about the relationship of oxysterols with neurodegenerative diseases such as AD, there are no direct data from humans to evaluate the relationship between oxysterols and MCI. The aim of this study was to evaluate plasma levels of 27–OHC, 24S–OHC, 7α–OHC and 7β–OHC in the elderly with and without MCI and attempt to establish potential relationships between these oxysterols and cognitive function.

## Methods

### Subjects and cognitive assessment

Seventy hospitalized subjects diagnosed as MCI and 140 controls with normal cognition were recruited from Xuan Wu Hospital in Beijing, China. The study design was ethically approved by the Ethics Committee of Capital Medical University (2013SY35). The process was explained for all subjects before the written informed consent was obtained. Controls were age- (±5 years), sex- and education- matched with MCI patients. The subjects with the history of a cerebrovascular event, malignant tumor and psychiatric illness or other neurological disease and statin or hypnotic sedative drugs abuse were excluded from the study. Cognitive function was evaluated by professional interviewers using the Mini–Mental State Examination (MMSE) [19] and Montreal Cognitive Assessment (MoCA) [20]. MMSE is commonly used to screen for dementia but insensitive to MCI while the MoCA was specially developed for detection of MCI [20]. All MCI patients should primarily satisfy the criteria of MCI including the following: (1) normal general cognitive function and absence of dementia that is sufficient to satisfy MMSE score of >19 for illiterate individuals, >22 for individuals with 1 to 6 years of education and >26 for individuals with 7 or more years of education; (2) mild impairment of cognitive functioning evaluated by MoCA score of ≤14 for illiterate individuals, ≤19 for individuals with 1 to 6 years of education and ≤24 for individuals with 7 or more years of education. If the subjects meet the above MCI criteria, they will visit a neurologist to make the final diagnosis.

### Demographic, clinical and anthropometric assessment

Demographics (age, gender, education, weight and height), lifestyle habits (current smoking status and drinking status), history of hypertension, coronary heart disease, diabetes and cerebrovascular disease were collected by self–reported questionnaire. Body mass index (BMI) was calculated as weight (kg)/height^2^ (m^2^). Current smoking status and drinking status were binary variables. Subjects were classified as smokers if they reported smoking three or more cigarettes a week for more than six months before enrollment and non-smokers if their cigarettes consumption was lower than this. Drinkers were identified by reporting alcohol consumption three or more times a week for more than six months before enrollment and non-drinkers lower than this.

### Laboratory measurements

The 0.6 mL tubes containing EDTA anticoagulant were used to collect fasting venous blood samples. The plasma samples were harvested after centrifugation at 3000 rpm for 10 min at 4 °C and stored frozen at −80 °C until measurement. The levels of plasma triglycerides (TG), total cholesterol (TC), high–density lipoprotein cholesterol (HDL–C), low-density lipoprotein cholesterol (LDL–C) and fasting blood glucose (FBG) were measured on a HITACHI 7600 analyzer. Aβ1-40 and Aβ1-42 plasma levels were evaluated by ELISA kit.

Plasma levels of oxysterols were measured using High Performance Liquid Chromatography–Mass Spectrometry (HPLC–MS) as described by Ines Burkard, et al. [21] with slight modifications. Briefly, 0.1 mL of plasma sample was transferred to a screw–capped vial and 100 ng of 19–hydroxycholesterol (19–OHC) was also added to the vial serving as internal standard. Alkaline hydrolysis was performed at 50 °C water bath for 2 h after adding 1.5 mL of 1 M ethanolic sodium hydroxide. Phosphoric acid (50 %) and 1 mL of phosphate buffer were added to the samples to adjust pH to 7. Supernatant was harvested after the centrifugation at 1000 g for 5 min and then applied to the C18 cartridges for solid–phase extraction. The eluted substances were dried at 30 °C and dissolved in 100 mL of methanol for future test. HPLC with an Angilent G1312B HPLC Pump and an Angilent C18 column (0.35 μm bead size; 4.6 × 250 mm) were used for the measurement of oxysterols. Quantification of oxysterols was performed using the multiple reaction monitoring (MRM) mode.

### Statistical analysis

The data were expressed by means ± standard deviations for normally distributed continuous variables, medians (interquartile ranges) for non–normally distributed continuous variables and frequencies (percentages) for categorical variables. Independent t–test and Mann Whitney *U* test were used for continuous variables and Chi–square test for categorical variables to compare differences between MCI and control groups. 27–OHC, 24S–OHC, 7α–OHC and 7β–OHC levels were classified into high and low levels by their medians. Univariate conditional logistic regression was used to evaluate the association between four oxysterols (treated as categorical variables) and MCI risk. Multivariate analysis was used to adjust demographic, clinical and anthropometric characteristics. Spearman rank correlation test was calculated to assess correlation coefficients. And *P* < 0.05 was considered statistically significant. All of the statistical analyses were performed using SPSS (version 18.0).

## Results

This study included 70 MCI patients (35 men and 35 women) and 140 controls with normal cognitive state (70 men and 70 women). Demographic and clinical characteristics of all the subjects were summarized in Table 1. Drinkers (*P* = 0.03), MoCA scores (*P* < 0.01), Aβ1-40 (*P* < 0.01) and Aβ1-42 (*P* < 0.01) were observed with significant differences between MCI and control group.

**Table 1:** Demographic and clinical characteristics of MCI patients and controls

	MCI		Controls		*P* value
	(*n* = 70)		(*n* = 140)		
Demographic and risk factors					
Age (y)	59	(61–72)	60	(62–69)	–
Male, %	50.0		50.0		–
Education (y)					–
≤ 9, (%)	44.3		44.3		
9 ~ 12, (%)	25.7		25.7		
≥ 12, (%)	30.0		30.0		
Smokers, %	32.9		22.9		0.12^a^
Drinkers, %	22.9		37.9		0.03^a^
Hypertension, %	41.4		41.4		1.00^a^
Coronary Heart Disease, %	7.1		8.6		0.72^a^
Diabetes, %	20.0		12.1		0.13^a^
Cerebrovascular Disease, %	2.9		2.9		1.00^a^
BMI (kg/m^2^)	24.42	±2.53	25.25	±3.78	0.10^b^
MCI screening					
MMSE scores	28	(27–29)	28	(27–30)	0.07 ^c^
MoCA scores	22	(19–23)	26	(25–28)	<0.01^c^
Laboratory					
FBG (mmol/L)	5.58	(5.20–6.25)	5.54	(5.09–6.14)	0.38^c^
TG (mmol/L)	1.38	(0.97–2.03)	1.41	(1.01–2.07)	0.69^c^
HDL-C (mmol/L)	1.4	(1.18–1.60)	1.4	(1.10–1.53)	0.71^c^
LDL-C (mmol/L)	2.57	±0.75	2.76	±0.74	0.09^b^
TC (mmol/L)	4.56	±1.00	4.73	±0.85	0.20^b^
Aβ1-40 (pg/mL)	992.08	±208.92	589.08	±214.39	<0.01^b^
Aβ1-42 (pg/mL)	795.01	±177.43	451.54	±209.08	<0.01^b^

^a^ Data presented as frequencies (percentages) were compared between 2 groups by using Chi-square test

^b^ Data presented as means ± standard deviations were compared between 2 groups by using the Student t–test

^c^ Data presented as medians (interquartile ranges) were compared between 2groups by using the Mann Whitney *U* test

The plasma levels of four oxysterols were present in Table 2. There was significant difference between the two groups regarding the plasma 27–OHC levels but no significant differences in 24S–OHC, 7α–OHC and 7β–OHC levels.

**Table 2:** Plasma levels of four oxysterols in MCI patients and controls (ng/mL)

Oxysterol	27–OHC	24S–OHC	7α–OHC	7β–OHC
MCI	74.23 (55.34–99.50)	44.55 (28.91–60.04)	47.48 (31.89–65.47)	51.20 (38.84–72.13)
(*n* = 70)				
Controls	56.48 (43.71–82.13)	44.69 (35.81–65.60)	45.57 (31.10–63.78)	52.07 (37.47–75.86)
(*n* = 140)				
*P* value	<0.01 ^a^	0.69 ^a^	0.68 ^a^	0.87 ^a^

^a^ Data presented as medians (interquartile ranges) were compared between 2 groups by using the Mann Whitney *U* test

Table 3 using univariate analysis showed that only high plasma level of 27-OHC was associated with MCI (OR = 3.21, 95 % CI: 1.76 ~ 5.85). Four oxysterols were classified into high and low levels by their medians.

**Table 3:** Odds ratio of MCI for oxysterols in univariate regression analysis

Oxysterols	Odds ratio and 95 % CI		*P* value
	Low level	High level	
27-OHC	Ref	3.21 (1.76–5.85)	<0.01
24S-OHC	Ref	0.93 (0.51–1.68)	0.80
7α-OHC	Ref	1.26 (0.66–2.41)	0.48
7β-OHC	Ref	0.89 (0.46–1.73)	0.74

The plasma levels of 27–OHC, 24S–OHC, 7α–OHC and 7β–OHC were classified into high and low levels by their medians

Table 4 showed the significant association between high plasma level of 27-OHC and MCI persisted even after adjustment (OR = 2.86, 95 % CI: 1.52 ~ 5.37).

**Table 4:** Results of univariate and multivariate regression analysis for 27-OHC

Model	Odds ratio and 95 % confidence interval		*P* value
	Low 27–OHC	High 27–OHC	
Unadjusted	Ref	3.21 (1.76–5.85)	<0.01
Adjusted^a^	Ref	2.86 (1.52–5.37)	<0.01

^a^Adjusted for demographic and risk factors

**Fig. 1:**
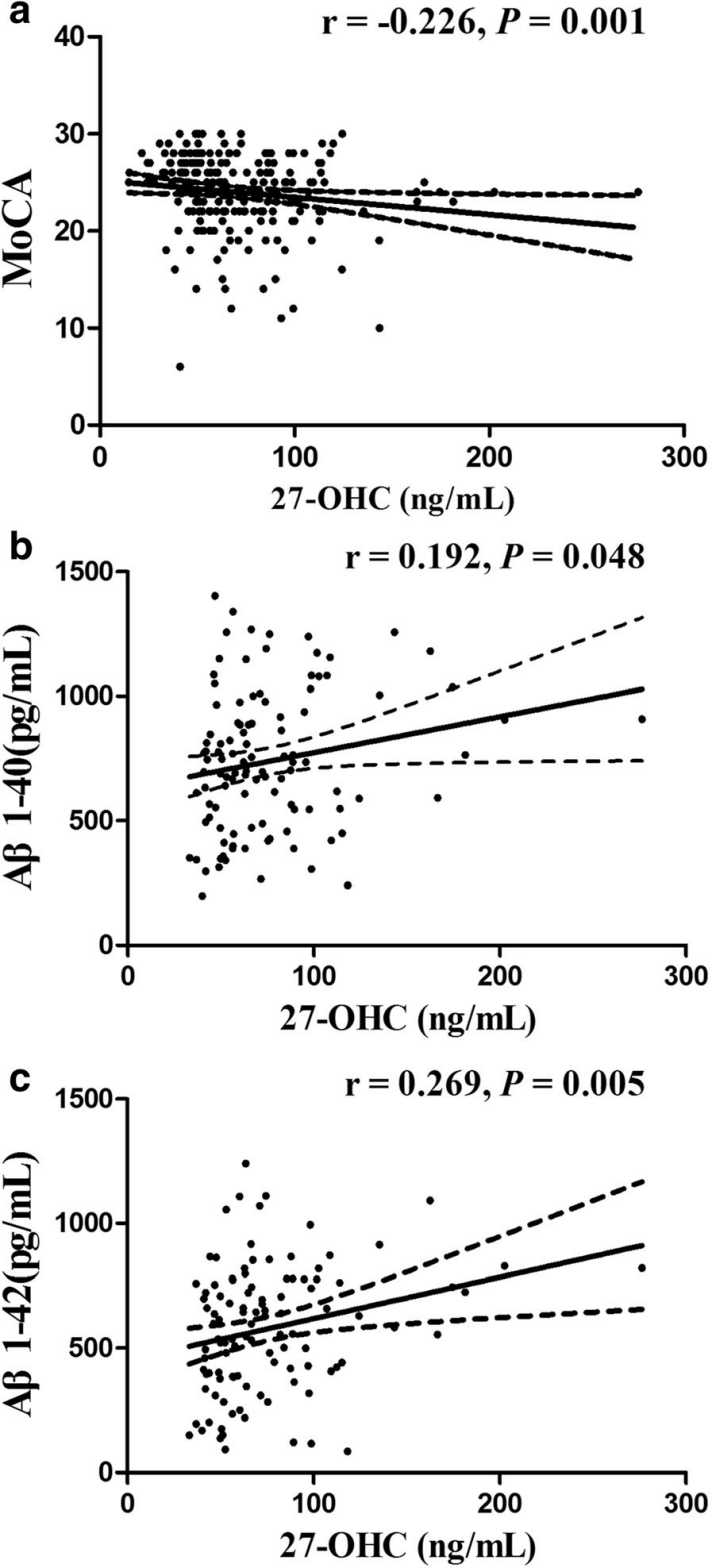
Correlations between 27-OHC vs.: **a** MoCA scores; **b** Aβ1-40 and **c** Aβ1-42 in cases and controls

Spearman correlation analyses showed that the plasma level of 27-OHC was positively correlated with that of Aβ1-40 and Aβ1-42 and negatively correlated with MoCA scores (Fig. 1a–c).

## Discussion

In these four oxysterols, 27–OHC is the major cholesterol metabolite in the circulation and mainly produced from cholesterol in periphery and synthesized in almost all cells by the cytochrome P-450 enzyme CYP27A1 located in the inner mitochondrial membranes. A significant association between high plasma level of 27-OHC and MCI was observed in this study. Our results were in accordance with the previous studies that described 27–OHC level in blood was negatively associated with cognitive performance in aging population and was significantly higher in AD patients [22, 23]. However, subjects with neurological diseases, such as AD or other types of dementia, were ruled out by exclusion criteria in our study for more reliable results prior to medication. There was also a negative correlation between plasma 27-OHC level and MoCA scores (r = − 0.269, *P* = 0.001). The negative correlation indicates that 27-OHC production in the blood is expected to increase with severity of cognitive impairment.

Previous research has indicated that subjects with high Aβ levels showed increased cognitive impairment [24]. Marwarhaet al [25] has found that 27-OHC induced 3-fold increase in Aβ1-42 and 1.5-fold increase in Aβ1-40 levels in the organotypic slices from rabbits. Moreover, Prasanthiet al [26] treated human neuroblastoma SH-SY5Y cells with 27-OHC and found a substantial increase in Aβ1-42 levels compared to untreated cells. Our study has also shown MCI patients had higher plasma Aβ1-40 and Aβ1-42 levels than controls with normal cognition (*P* <0.01). Simultaneously, a good positive correlation between plasma levels of Aβ1-42 and 27-OHC (r = 0.269, *P* = 0.005) and a weak but significant correlation of plasma 27-OHC with Aβ1-40 levels (r = 0.192, *P* = 0.048) were also observed, supporting the hypothesis that 27-OHC may enhance circulating amyloid production and increase the risk of cognitive impairment.

Despite that, studies analyzing the associations between plasma 27–OHC level and cognitive decline yielded conflicting results. Timothy M. Hughes et al. [27] recently found that the increase of plasma 27–OHC levels was related to cerebrovascular disease prior to cognitive decline over many years of follow–up. However, it lacked MRI results for cerebrovascular disease when the volunteers were diagnosed of AD or MCI in follow–up. Thus, the question arises whether cerebrovascular disease is the injury factor for cognitive status. In addition, a case–control study has shown that the ratio of 27–OHC to total circulating cholesterol (27–OHC/Chol) level is lower in AD and MCI patients than that in controls [28]. There is possibility that oxysterols and cholesterol compete for space within the lipoproteins and they have different scales on space within the lipoprotein, absolute levels of plasma 27–OHC may be higher in MCI compared to controls despite of the decrease of 27–OHC/Chol.

On the other hand, in the brain, cholesterol is removed by conversion to 24S–OHC via CYP46A1 enzyme, which is primarily expressed in neurons. We found no significant difference in 24S–OHC level in plasma between MCI patients and control group. In contrast to the former research, they observed significantly elevated or declined plasma levels of 24S–OHC in AD, vascular disease (VaD) and MCI participants [29, 30]. These conflicting findings may result from study population with different time after being diagnosed with MCI. The late MCI patients with the loss of neuronal cells had decreased level of 24S–OHC whereas the early MCI patients were characterized by the increase of 24S–OHC probably as a consequence of the released cholesterol caused by the myelin disruption [31].

Unlike 27–OHC and 24S–OHC, 7β–OHC is generated by non–enzymatic oxidation whereas 7α–OHC is generated by both non–enzymatic and enzymatic oxidation that is catalyzed by CYP7A1 [32]. The effects of 7α–OHC and 7β–OHC on cognitive function are less known. MCI falls in between normal forgetfulness and AD. It is accepted that early intervention in MCI including decreasing the risk factors is useful. Our findings has offered some valid epidemiological evidence to reveal the role of 27-OHC in the pathogenesis of MCI, which may provide new insights into the prevention of AD. Some experiments in cell cultures and animals have suggested increased levels of 27-OHC may trigger or accelerate progression of AD or MCI through a variety of mechanisms. However, efforts to find out the role of 27-OHC in AD or MCI are still necessary by further human studies. The strengths of our study was a matched case–control study after adjustment for confounders and based on standardized epidemiological methods. Additionally, we enrolled MCI patients without medication as the target population in order to more directly investigate the relationship with risk factors than AD patients and take preventive measures in the preclinical stage of AD. However, it was a case–control study that can not establish the timeline of exposure to disease outcome, prospective cohort studies are also needed to further evaluate the role of oxysterols in AD or MCI.

## Conclusions

In conclusion, our findings suggested plasma level of 27-OHC was significantly higher in MCI patients than controls with normal cognition and the increased plasma level of 27-OHC was significantly associated with MCI. Prospective cohort studies and experiments in vitro are needed to further evaluate the potential role of 27-OHC and other oxysterols in involvement in AD or MCI.

## Abbreviations

24S–OHC: 24S–hydroxycholesterol
27–OHC: 27–hydroxycholesterol
7α–OHC: 7α–hydroxycholesterol
7β–OHC: 7β–hydroxycholesterol
AD: Alzheimer’s disease
HPLC–MS: High Performance Liquid Chromatography–Mass Spectrometry
MCI: Mild cognitive impairment
